# Metagenomic Analyses of Microbial and Carbohydrate-Active Enzymes in the Rumen of Dairy Goats Fed Different Rumen Degradable Starch

**DOI:** 10.3389/fmicb.2020.01003

**Published:** 2020-05-20

**Authors:** Jing Shen, Lixin Zheng, Xiaodong Chen, Xiaoying Han, Yangchun Cao, Junhu Yao

**Affiliations:** College of Animal Science and Technology, Northwest A&F University, Yangling, China

**Keywords:** metagenomics, microbiome, CAZymes, rumen degradable starch, dairy goats

## Abstract

The objective of this study was to investigate the effects of different dietary rumen degradable starch (RDS) on the diversity of carbohydrate-active enzymes (CAZymes) and Kyoto Encyclopedia of Genes and Genomes Orthology functional categories to explore carbohydrate degradation in dairy goats. Eighteen dairy goats (second lactation, 45.8 ± 1.54 kg) were divided in three groups fed low RDS (LRDS), medium RDS (MRDS), and high RDS (HRDS) diets. The results showed that, HRDS treatment group significantly decreased the ruminal pH (*P* < 0.05), and increased the propionate proportion (*P* < 0.05), fumarate and succinate concentrations (*P* < 0.05), trended to increase lactate concentration (*P* = 0.50) compared with LRDS group. The relative abundance of acetogens, such as family Clostridiaceae and Ruminococcaceae, genera *Clostridium* and *Blautia* were higher in HRDS than LRDS feeding goats. The GH9 family (responsible for cellulose degradation) genes were lower in HRDS than MRDS diet samples, and mainly produced by Prevotellaceae, Ruminococcaceae, and Bacteroidaceae. Amylose (EC3.2.1.3) genes under HRDS treatment were more abundant than under LRDS treatment. However, the abundance of GH13_9 and CBM48 (responsible for starch degradation) were reduced in HRDS group indicating the decreased binding activity from catalytic modules to starch. This study revealed that HRDS-fed dairy goats had decreased CAZymes, which encode enzymes degrade cellulose and starch in the dairy goats.

## Introduction

The rumen is recognized as a natural bioreactor for highly efficient carbohydrates degradation (Codron and Clauss, 2010), because it harbors large numbers of microorganisms, including bacteria, archaea, fungi and protozoa. The bacteria are most abundant with an estimated population density of 10^10–11^ mL^–1^ of rumen fluid, followed by archaea (10^8–9^ mL^–1^), ciliate protozoa (10^6^ mL^–1^) which contribute up to half of the rumen microbial biomass due to their large size, and fungi with 10^6^ mL^–1^ contributing less than 8% to total biomass (Wang et al., 2017). Rumen microorganisms produce a series of enzymes known as carbohydrate-active enzymes (CAZymes) which break down plant polysaccharides into their oligomers and monomers, then were fermented to yield volatile fatty acids (VFAs) (Vincent et al., 2014). CAZymes control the diversity of complex carbohydrates involved in their assembly (glycosyltransferases, GTs) and their breakdown (glycoside hydrolases, GHs; polysaccharidelyases, PLs; carbohydrate esterases, CEs). Furthermore, the non-enzymatic species carbohydrate-binding modules (CBMs) can increase the catalytic activity by specially binding polysaccharides (Boraston et al., 2004; Jones et al., 2018). These CAZymes cooperatively contribute to dietary carbohydrate deconstruction, such as cellulose, hemicellulose, and starch (Lim et al., 2013; Kala et al., 2017).

For ruminants, starch an important energy source is often used to improve rumen fermentation, optimizing digestion of carbohydrates and increasing protein flow to the small intestine. Compared with corn starch, wheat starch has higher amylopectin content, and less bound with insoluble protein, resulting in more accessible to enzymatic hydrolysis in wheat starch (Luis et al., 2016). Increasing dietary rumen degradable starch (RDS) can increase the potential risk for sub-acute ruminal acidosis in goats by decreasing the ruminal pH (Gozho et al., 2007; Li et al., 2014b). Meanwhile, increasing RDS can decrease the ratio of acetate to propionate, changing the abundance of cellulolytic bacteria and amylolytic bacteria (Li et al., 2014a, b). However, these studies only focused on the rumen fermentation and microbial community composition and did not provide information on microbial metabolic functions.

Metagenomics are approaches that can be used to study functional aspects of the microbial community at the genomic level, specially the microbial metabolic functions. To date, most research has been conducted on CAZymes and digestive microbiota in ruminants (Dai et al., 2015; Jose et al., 2017; Kala et al., 2017), but few studies have evaluated the effects of different RDS on the diversity of CAZymes and taxonomic profile of rumen microbial communities by metagenomics analyses in dairy goats. Therefore, this study analyzed CAZymes and Kyoto Encyclopedia of Genes and Genomes (KEGG) Orthology (KO) functional categories to explore carbohydrate degradation under different RDS in dairy goats.

## Materials and Methods

### Ethics

In this study, all animal procedures were approved by the Institutional Animal Care and Use Committee of Northwest A&F University.

### Animals and Diets

Briefly, eighteen Guanzhong lactating goats (second lactation) of 45.8 ± 1.54 kg body weight were paired and randomly allocated to three groups according to body weight and milk yield after receiving a basal diet with a forage-to-concentration ratio of 45:55 for 2 weeks before this study. The three treatments diets were formulated to be isoenergetic, isonitrogenous, iso-starch, and different in the RDS through replacing corn with wheat: low RDS (LRDS = 20.52%), medium RDS (MRDS = 22.15%) and high RDS (HRDS = 24.88%). The composition and nutrient contents of the diets were given in Table 1. During the experimental period, the animals were fed separately twice daily at 0800 and 1630 h, and the water was available *ad libitum*. All goats were milked twice daily at 0800 and 1600 h. The feed intake and milk composition and yield for those goats have been published elsewhere (Zheng et al., 2020).

**TABLE 1:** The composition and ingredients of experimental diets.

**Item**	**Treatments**		
	**LRDS**	**MRDS**	**HRDS**
**Ingredient, (% of DM)**			
Alfalfa hay	17.50	17.50	17.50
Corn silage	27.50	27.50	27.50
Corn	40.00	23.50	8.00
Wheat	–	18.00	36.00
Soybean meal	13.00	7.60	5.00
Corn gluten meal	–	1.90	2.00
Wheat bran	–	2.00	2.00
Calcium phosphate	0.25	0.25	0.25
Limestone	0.75	0.75	0.75
Salt	0.50	0.50	0.50
Vitamin-mineral mix^1^	0.50	0.50	0.50
**Nutrient composition^2^**			
DM,%	50.58	50.27	50.21
ADF%	18.88	18.58	18.22
NDF%	34.38	33.76	32.90
CP%	16.40	16.71	16.59
Starch%	27.66	27.54	28.58
RDS%	20.52	22.15	24.88

*^1^Vitamin-mineral mix (per kilogram): 450 mg of nicotinic acid, 600 mg of Mn, 950 mg of Zn, 430 mg of Fe, 650 mg of Cu, 30 mg of Se, 45 mg of I, 20 mg of Co, 800 mg of vitamin E, 45,000 IU of vitamin D, and 120,000 IU of vitamin A. ^2^DM, dry matter; ADF, acid detergent fiber; NDF, neutral detergent fiber; CP, crude protein; RDS, rumen degradable starch. Assuming a rumen outflow rate of 6%/h.*

### Sample Collection and Analysis

All goats were killed under anesthesia 3 h after morning feeding at day 36. Rumen fluid was collected and strained through four layers of sterile cheesecloth and pH was immediately measured by using a mobile pH meter (Ohaus Instruments Co., Ltd., Shanghai, China). Collected rumen fluid was kept in lipid nitrogen until being stored at −80°C. The detection of ruminal VFAs (acetate, propionate, butyrate, valerate, isobutyrate, and isovalerate) concentrations referred to the previous study (Li et al., 2014b). Briefly, 4 mL rumen liquid was mixed with 1 mL metaphosphoric acid (250 g/L), then were centrifuged for 15 min at 10,000 × *g* at 4°C. Two milliliters of the supernatant were mixed with 200 μL crotonic acid (10 g/L) and then filtered through a 0.45 μm filter. The ruminal VFAs were separated and quantified by Agilent 7820A GC system. The chromatograms of GC for analytical standard and ruminal samples of the study groups were shown in Supplementary Figure S1.

The detailed procedures of fumarate, succinate, and lactate concentrations analysis referred to the previous study (Shurubor et al., 2016). Briefly, 550 μL rumen fluid was mixed with 200 μL ice-cold 10% perchloric acid. The acid-treated rumen fluid was then vortexed for 1 min and incubated on ice for 10 min. The precipitated protein was removed by centrifugation at 14, 000 × *g* for 20 min at 4°C. The protein-free supernatant was transferred to another polypropylene tube and a second centrifugation was performed under the same conditions. The protein-free extract was analyzed by Agilent 1260 high-performance liquid chromatography system. The chromatograms of HPLC for analytical standard and ruminal samples of the study groups were shown in Supplementary Figure S2.

### DNA Extraction, Metagenome Library Preparation and Sequencing

The total DNA was extracted by CTAB/SDS method (Kumar et al., 2014). The DNA concentration was determined by using a Nanodrop 1000 (Thermo Fisher Scientific, Wilmington, DE, United States), and the purity was monitored on 1% agarose gels. The DNA was stored at −80°C until further processing.

DNA extract was fragmented to an average size of about 300 bp using Covaris M220 (Gene Company Limited, China) for paired-end library construction. Paired-end library was constructed using TruSeq^TM^ DNA Sample Prep Kit (Illumina, San Diego, CA, United States). Adapters containing the full complement of sequencing primer hybridization sites were ligated to the blunt-end of fragments. Paired-end sequencing was performed on Illumina HiSeq4000 platform (Illumina Inc., San Diego, CA, United States) at Majorbio Bio-Pharm Technology Co., Ltd., (Shanghai, China) using HiSeq 3000/4000 PE Cluster Kit and HiSeq 3000/4000 SBS Kit according to the manufacturer’s instructions^1^.

### Sequence Quality Control and Genome Assembly

Adapter sequence were stripped from the 3′ and 5′ end of paired end Illumina reads using SeqPrep (Verision 1.1). Low-quality reads (length < 50 bp or with a quality value < 20 or having N bases) were removed by Sickle (Verision 1.33). Reads were aligned to the Capra hircus genome by BWA (Version 0.7.9a) and any hit associated with the reads and their mated reads were removed. Metagenomics data were assembled by multiple_ megahit using Megahit (Version 1.1.2), which makes use of succinct de Bruijn graphs. Contigs with the length being or over 300 bp were selected as the final assembling result, and then the contigs were used for further gene prediction and annotation. The summary statistics for multiple_megahit was shown in Supplementary Table S1. The scaftigs length distribution was shown in Supplementary Figure S3.

### Gene Prediction, Taxonomy, and Functional Annotation

Open reading frames (ORFs) from each assembled contig were predicted using MetaGene (Noguchi et al., 2006). The predicted ORFs with length being or over 100 bp were retrieved and translated into amino acid sequences using the NCBI translation table. All predicted genes with a 95% sequence identity (90% coverage) were clustered using CD-HIT (Version 4.6.1) (Fu et al., 2012), the longest sequences from each cluster were selected as representative sequences to construct non-redundant gene catalog. Reads after quality control were mapped to the representative sequences with 95% identity using SOAPaligner Version 2.2.1 (Li et al., 2008), and gene abundance in each sample were evaluated via reads per kilobase per million mapped (RPKM). Representative sequences of non-redundant gene catalog were aligned to NCBI NR database (Version: 2017-12-26) using Diamond (blsatp 2.3.0 with default parameter values, except e-value ≤ 1e-5) for taxonomic annotations using Best-hit method (Altschul et al., 1997). The KEGG annotation was conducted using Diamond against the Kyoto Encyclopedia of Genes and Genomes database (Version: 2018-07-30) (Xie et al., 2011) with an e-value cutoff of 1e-5. Carbohydrate-active enzymes annotation was conducted using hmmscan (Version 3.1b2) against CAZy database Version 6.0 with an e-value cutoff of 1e-5.

### Pyrosequencing Data Accession Number

The Illumina sequencing raw data for our samples have been deposited in the NCBI Sequence Read Archive (SRA) under accession numbers: PRJNA622867.

### Statistical Analysis

The data of rumen metabolites were presented as mean ± SE and the difference between two groups was analyzed by Student’s *t* test using the SPSS software (version20.0; Chicago, IL, United States). Taxonomic and functional data were analyzed on the online platform of Majorbio Cloud Platform^2^. Differential abundance of phylum, family, genus, CAZymes and KO modules was tested by Wilcoxon test using stats R package in R software (version 3.3.1). The Differences were statistically significant at *P* < 0.05 or a tendency of difference at 0.05 ≤ *P* < 0.10.

## Results

### Ruminal Parameters

**FIGURE 1:**
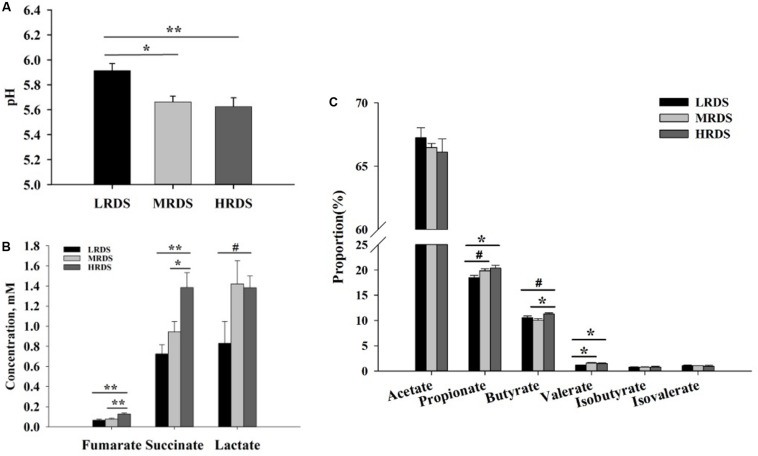
Effect of dietary RDS on the rumen fermentation parameter. **(A)** Ruminal pH. **(B)** Concentrations of fumarate, succinate, and lactate. **(C)** Proportion of VFA. (*n* = 6 per group) *P < 0.05, **P < 0.01, ^#^0.05 ≤ *P* < 0.10.

The effect of dietary RDS on the rumen fermentation parameter were shown in Figure 1. The ruminal pH was significantly decreased in MRDS (5.66 ± 0.046, *P* < 0.05) and HRDS (5.62 ± 0.072, *P* < 0.01) groups, compared to LRDS group (5.91 ± 0.057). The concentration of fumarate was significantly increased in HRDS group (0.13 ± 0.01), compared to LRDS (0.06 ± 0.01, *P* < 0.05) and MRDS (0.08 ± 0.01, *P* < 0.05) groups. The concentration of succinate was significantly increased in HRDS group (1.38 ± 0.15), compared to LRDS (0.73 ± 0.09, *P* < 0.05) and MRDS (0.94 ± 0.10, *P* < 0.05) groups. The concentration of lactate trended to be increased in HRDS group (1.38 ± 0.12, *P* = 0.05), compared to LRDS group (0.83 ± 0.22). The propionate proportion was significantly increased in HRDS group (20.34% ± 0.59%), compared to LRDS group (18.43% ± 0.45%, *P* < 0.05). The butyrate proportion was significantly increased in HRDS group (11.28% ± 0.21%), compared to MRDS group (10.06% ± 0.28%, *P* < 0.05). The valerate proportion was significantly increased in HRDS group (1.48% ± 0.09%), compared to LRDS group (1.17% ± 0.03%, *P* < 0.05).

### Ruminal Microbial Composition

**FIGURE 2:**
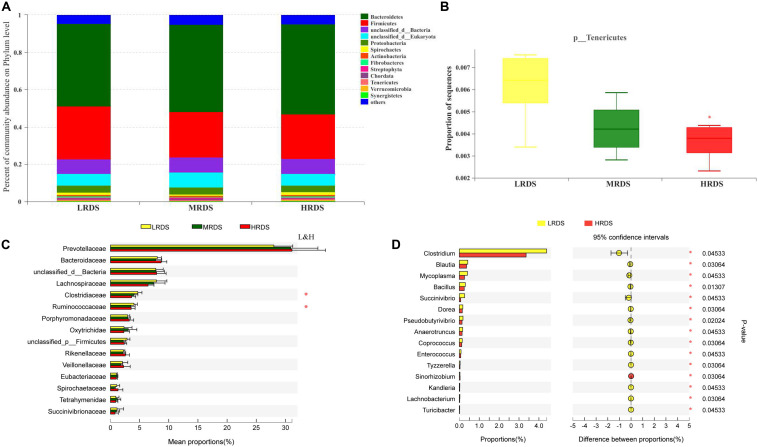
**(A)** Stacked bar graphs showing relative abundance of dominant phylum. **(B)** Relative abundance of *Tenericutes* were compared. **(C)** Top 15 family were compared between each two groups. **(D)** Top 15 genera that different between LRDS and HRDS group. (*n* = 6 per group) *P < 0.05.

Metagenome sequencing of the total DNA from 18 rumen samples generated approximately 248 gigabases of raw sequences data. At the phyla level, Bacteroidetes, Firmicutes, Proteobacteria, and Spirochetes were the dominant phyla (Figure 2A). Compared with LRDS group, HRDS group significantly decreased the relative abundance of Tenericutes (*P* < 0.05) (Figure 2B). At the family level, Prevotellaceae, Bacteroidaceae, Lachnospiraceae, Clostridiaceae, and Ruminococcaceae were the dominant family. Compared with LRDS group, HRDS group significantly decreased the relative abundance of Clostridiaceae, and Ruminococcaceae (Figure 2C). At the genus level, compared with LRDS group, HRDS group significantly decreased the relative abundance of *Clostridium*, *Blautia*, *Mycoplasma*, *Bacillus*, *Succinivibrio*, and *Pseudobutyrivibrio* (Figure 2D).

### Functions of the Rumen Microbiome

#### Carbohydrate Genes Related to Carbohydrate Degradation Pathway

**FIGURE 3:**
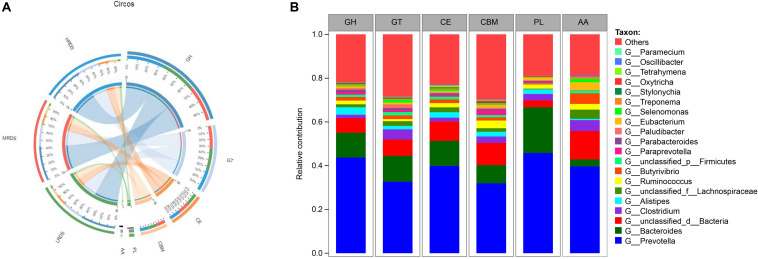
**(A)** Percent of CAZymes in each group. **(B)** Percent contributions of CAZymes from the major microbial communities in dairy goats. GH stands for glycoside hydrolase, GT for glycosyltransferase, PL for polysaccharide lyase. CE for carbohydrate esterases, CBM for carbohydrate-binding module, and AA for auxiliary activity.

Rumen microorganisms produce a range of enzymes collectively known as CAZymes, including cellulases, hemicellulases, and amylases attributed to dietary degradation. To specifically explore the microbial potential for dietary degradation in ruminal metagenomes, we screened for CAZymes in the assembled contigs. CAZymes were determined to belong to different classes (GHs, GTs, CEs, CBMs, PLs, and AAs) were shown in Figure 3A. Among these six classes of CAZymes families, GHs and GTs were the most families, and no differences were found among the treatments. Phylogenetic analysis of CAZyme contigs showed that *Prevotella*, *Bacteroides*, and *Clostridium* primarily contributed CAZyme-encoding gene fragments of the GH, GT, CE, CBM, PL, and AA families in the dairy goats’ rumen metagenome, followed by *Aliptipes*, *unclassified_f_Lachnospiraceae*, and *Ruminococcus* (Figure 3B).

**FIGURE 4:**
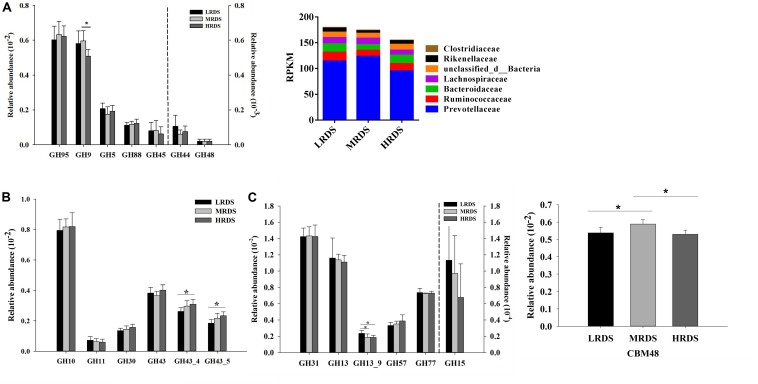
**(A)** Comparisons of the gene abundance of the GH family gene-coded cellulase. Phylogenetic distribution of sequences in GH9 assigned to the identified family. **(B)** Comparisons of the gene abundance of the GH family gene-coded hemicellulase. **(C)** Comparisons of the gene abundance of the GH family gene-coded amylase. Comparisons of the gene abundance of CBM48. (*n* = 6 per group) *P < 0.05.

To further provide support for the pivotal carbohydrate biodegradation process, we compared the GH families responsible for cellulose, hemicellulose, and starch degradation. The CBM domain helps in binding of a CAZyme to its carbohydrate substrate, thereby facilitating the enzyme’s activity. In the present study, five GH families (GH97, GH9, GH5, GH88, and GH45) were mainly found to be associated with cellulolytic functions (Figure 4A). The abundances of GH9 was significantly decreased in HRDS group compared to LRDS group (*P* < 0.05). Notably, the GH9 genes were mainly phylogenetically assigned to Prevotellaceae, Ruminococcaceae, and Bacteroidaceae at the family level (Figure 4A). Five GH families (GH10, GH11, GH30, and GH43) were mainly found to be associated with hemicellulolytic functions. In the present study, we found that the abundance of GH43_4 and GH43_5 genes were significantly increased in HRDS group compared to LRDS group (*P* < 0.05) (Figure 4B). Four GH families (GH31, GH13, GH57, and GH77) were mainly found to be associated with starch degradation, the abundance of GH13_9 was significantly decreased in MRDS (*P* < 0.05) and HRDS (*P* < 0.05) groups compared with LRDS group (Figure 4C). CBM48-GH13 is a documented architecture for starch-degrading enzymes (Machovic and Janecek, 2008). We found that MRDS group significantly increased the abundance of CBM 48 compared to LRDS (*P* < 0.05) and HRDS group (*P* < 0.05) (Figure 4C).

**FIGURE 5:**
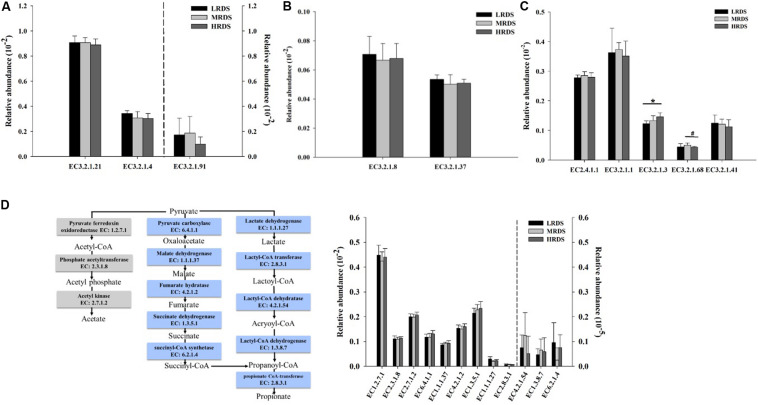
Comparisons of the relative abundance of KO enzymes for cellulase **(A)**, xylanase **(B)**, and amylase **(C)**. **(D)** Metabolic routes for propionate and acetate production by direct conversion from pyruvate. Comparisons of the relative abundance of KO enzymes related to the propionate and acetate production pathway. (*n* = 6 per group) *P < 0.05, ^#^0.05 ≤ *P* < 0.10. EC 3.2.1.21, β-glucosidases; EC3.2.1.4, endoglucanases; EC3.2.1.91, exoglucanases; EC3.2.1.8, endoxylanases; EC3.2.1.37, 1,4-β-xylosidase; EC2.4.1.1, starch phosphorylase; EC3.2.1.1, α-amylase; EC3.2.1.3, amyloglucosidase; EC3.2.1.68, isoamylase; EC3.2.1.41, pullulanase.

The abundance of the KO genes related to carbohydrates degradation was compared between the treatments. The relative abundances of the cellulases including endoglucanases, exoglucanases, and β-glucosidases; hemicellulases including endoxylanases, exoxylanases, and 1,4-β-xylosidase; and amylase including α-amylase, amyloglucosidase, starch phosphorylase, isoamylase, and pullulanase were shown in Figure 5. Among the gene encoding enzymes, HRDS group significantly increased the relative abundance of the amyloglucosidase (EC3.2.1.3) compared to LRDS group (*P* < 0.05). The relative abundance of isoamylase (EC 3.2.1.68) genes were trended to decrease in HRDS compared with MRDS group (*P* = 0.066).

#### The Fermentation Pathways From Pyruvate Into Acetate and Propionate by Microorganisms

Many gene coding enzymes participate in the acetate and propionate fermentation pathways by rumen microorganisms. The fermentation pathways from pyruvate to propionate include succinate pathway and acrylate pathway. The genes encoding pyruvate fermentation to acetate and propionate were compared between the treatments. Among these encoding enzymes, no differences were found among the treatments (Figure 5D).

## Discussion

Ruminal pH and its daily fluctuation characteristics are influenced by rumen degradable carbohydrates. Crystal pattern, granule size and shape, amylose and amylopectin content, presence of a protein matrix are the main factors characterizing starch for each plant species (French, 1973). Compared with corn starch, wheat starch has higher amylopectin content, and less bound with insoluble protein, resulting in more accessible to enzymatic hydrolysis in wheat starch (Li et al., 2014a; Luis et al., 2016). In the current study, we conduct higher RDS by replacing corn with wheat. We have reported the effect of dietary RDS on milk production and found that high RDS diets significantly decreased the milk fat composition and yield in the dairy goats (Zheng et al., 2020). The milk fat synthesis in mammary gland can be affect by rumen metabolites that derived from rumen microorganisms, such as acetate, butyrate, *trans* fatty acids, and so on. In the present study, we analyzed the rumen fermentation, and the rumen microbial metabolic functions by metagenomics analyses to explore carbohydrate degradation under different RDS in dairy goats.

Previous studies reported that wheat decreased the ruminal pH because of the higher degradability of starch in wheat (Li et al., 2014b). The HRDS and MRDS group significantly decreased the ruminal pH, which is consistent with previous study (Li et al., 2014b). The higher proportion of propionate in HRDS group indicated that more starch was fermented in the rumen compared with the LRDS group. The higher concentration of fumarate, succinate, and lactate indicated that HRDS feeding enhanced the succinate pathway and acrylate pathway to produce propionate. The pH of lactate is lower than that of the VFA, can decrease the ruminal pH.

Previous studies showed that some taxa of Clostridiales, Ruminococcaceae, and *Blautia* contain acetogens (Yang et al., 2016; Mi et al., 2018). In the present study, order Clostridiales, family Clostridiaceae and Ruminococcaceae, genera *Clostridium* and *Blautia* were significantly lower in the HRDS group than in the LRDS group, which might indicate that HRDS feeding goats decrease the capacity to produce acetate.

Accumulated rumen metagenomic studies have shown physical functional genes in rumen samples, which provided a way to evaluate the function of rumen microorganisms (Hess et al., 2011; Madiajagan, 2015). Metagenomic analysis of CAZyme-encoding gene abundance and KEGG pathways gene abundance could help characterize carbohydrate degradation in rumen of dairy goats. The abundance of CAZyme-encoding genes and KO genes related to fiber and starch degradation were analyzed in rumens in response to different dietary RDS level. Rumen metagenomic analysis showed that GH families were most abundance in dairy goats, which was consistent with previous studies (Berlemont et al., 2015; Stewart et al., 2018). The GH families comprise a large number of enzymes involved in the degradation of polysaccharides such as cellulose, hemicellulose, starch, and chitin (Stewart et al., 2018). The high abundant of GHs showed the capacity of dairy goat rumen to break down plant cell wall polysaccharides.

Starch is the main energy component used in ruminants feed to improve ruminal fermentation. In rumen, starch is degraded mainly by the α-1,4 and α-1,6 amylases produced by rumen microorganisms to release different oligosaccharides. Previous study showed that wheat-induced SARA don’t affect effect ruminal degradability (ERD) of corn starch, but significantly decrease the rapidly degradable fraction of corn starch (Li et al., 2014a). We found that HRDS group significantly increased the amyloglucosidase (EC3.2.1.3) which can hydrolyze both α-1,4 and α-1,6 glycosyl, and produce glucose. Regarding the CAZymes, dramatically, family GH13 at high abundance is known as an α-amylase family that binds and degrades starch (Zhang et al., 2017; Sophie et al., 2018). CBM48-GH13 is a documented architecture for starch-degrading enzymes (Machovic and Janecek, 2008). The family CBM48 contains the putative starch-binding domains present in the four enzyme specificities from the α-amylase enzyme family: pullulanase (GH13_12, GH13_13, GH13_14), isoamylase (GH13_11), maltooligosyl trehalohydrolase (GH13_10), as well as the glycogen branching enzyme and the starch branching enzyme (GH13_8, GH13_9). In the present study, we found that HRDS significantly decreased the GH13_9 and CBM48 genes abundance. In the current study, the relative abundance of isoamylase (EC 3.2.1.68) genes were trended to decrease in HRDS compared with LRDS group. From these, we can speculate that HRDS group increased the RDS because of the easily accessible to enzymatic hydrolysis, but decrease the starch branching enzyme by altering the binding activity from catalytic modules to starch substrates. Further studies are needed to explore the mechanisms for the decreased binding activity.

During cellulose degradation, the cellulose fibrils are attacked at the ends of the chain by exoglucanases (EC3.2.1.91) that are generally from GH families 6, 7, and 48 (Morais and Mizrahi, 2019). The endoglucanases (EC 3.2.1.4) cleave the cellulose chain internally that are from GH families 5, 6, 7, 9, and 45 (Morais and Mizrahi, 2019). In the present study, we found that the genes that encode cellulases mainly belonged to GH95, GH9, GH5, GH88, and GH45 families, which were also determined by previous studies (Patel et al., 2014; Solden et al., 2018; Wang et al., 2019). Among these, GH95 and GH9 were most abundance, GH9 is well known with endoglucanase activity (Naas et al., 2014; Maharjan et al., 2018). The relative abundance of GH9 was decreased in high dietary concentrate-to-forage ration (Wang et al., 2019). In consistent with that, we found the abundance of GH9 family was lower under HRDS treatment. At the family level, the GH9 genes were mainly phylogenetically assigned to Prevotellaceae, Ruminococcaceae, and Bacteroidaceae. Meanwhile, Ruminococcaceae relative abundance was lower under HRDS treatment than LRDS treatment. Ruminococcaceae is the mainly cellulolytic bacteria. Cellulolytic bacteria are generally believed to be sensitive to ruminal pH, and a low pH compromises their growth (Khafipour et al., 2009). We can speculate that HRDS decreased the cellulase activity by decreasing the endoglucanases.

The second major component of the plant cell wall is hemicellulose. Hemicellulose is comprised of virous polysaccharides, with xylan being the most abundant. Xylanases are generally from GH families 10, 11, and 30 for the endoxylanases (EC3.2.1.8: cleaving the main chain internally) and 43 for exoxylanases (EC3.2.1.92: cleaving at the chain ends) (Morais and Mizrahi, 2019). In the present study, we found that the abundance of GH43_4 and GH43_5 genes were significantly increased in HRDS group compared to LRDS group. The corn bran xylans, which have the higher number and complexity of the side chains are more recalcitrant to breakdown by know xylan-degrading enzyme system than wheat flour xylans (Rogowski et al., 2016). These may explain the increment of the abundance of GH43_4 and GH43_5 in HRDS group which have higher wheat proportion compared with LRDS group.

The abundance of CAZyme-encoding genes and KO genes related to fiber and starch degradation indicate the degradation from fiber and starch to glucose, which in turn was fermented to VFA by microorganisms. The fermentation processes from glucose to the acetate and propionate are catalyzed by various enzymes. No differences in the abundance of these enzymes were observed at the metagenome level between groups. Therefore, the increased RDS content and decreased abundances of GH9 and acetogens altered the rumen fermentation in HRDS feeding goats. Altogether, these findings indicate that high RDS diets altered the composition and function of ruminal microbiota.

## Conclusion

In conclusion, this study investigated changes in rumen fermentation, microbial composition variation, and CAZyme-encoding genes and KO genes related to fiber and starch degradation in response to the dietary RDS in dairy goats. We revealed that dietary RDS significantly affect rumen fermentation and carbohydrate degradation. The HRDS group decreased ruminal pH, then inhibited dominant acetogens, and the genes abundance of endoglucanase. The HRDS group increased the propionate proportion by increased RDS content and the genes abundance of amylase, but decreased the starch branching enzyme by altering the binding activity from catalytic modules to starch substrates. Therefore, this study can enhance our understanding of starch and cellulose degradation in response to the changes of dietary RDS in the rumen of dairy goats, which contributes to the improvement of starch utilization in ruminant nutrition.

## Data Availability Statement

The raw data supporting the conclusions of this article will be made available by the authors, without undue reservation, to any qualified researcher.

## Ethics Statement

The animal study was reviewed and approved by the Institution Animal Care and Use Committee of the Northwest A&F University (protocol number NWAFAC1008).

## Author Contributions

JS, LZ, XH, and JY designed the research. JS, LZ, XH, and XC performed the research and analyzed the data. JS and JY wrote the manuscript. All authors taken part in the revision of the manuscript, read and approved the final version of the manuscript.

## Conflict of Interest

The authors declare that the research was conducted in the absence of any commercial or financial relationships that could be construed as a potential conflict of interest.

## Footnotes

1 www.illumina.com

2 www.majorbio.com
