# Supplementary material for: Metagenomic Analyses of Microbial and Carbohydrate-Active Enzymes in the Rumen of Dairy Goats Fed Different Rumen Degradable Starch
Source: Front Microbiol. 2020 May 20;11:1003. doi: 10.3389/fmicb.2020.01003 (PMC7251062; doi:10.3389/fmicb.2020.01003)
Supplement: Supplementary file 1 [file Data_Sheet_1.docx]

**Running title:** Microbial and Carbohydrate-Active Enzymes in Rumen of Goats

**Title:** Metagenomic analyses of microbial and carbohydrate-active enzymes in the rumen of dairy goats fed different rumen degradable starch

**Authors:** Jing Shen, Lixin Zheng, Xiaodong Chen, Xiaoying Han, Yangchun Cao, Junhu Yao*

***Corresponding author:**

Junhu Yao: [yaojunhu2008@nwsuaf.edu.cn](mailto:yaojunhu2008@nwsuaf.edu.cn)

**Supplementary files:**

**Supplementary Table S1** The summary statistics for Multiple_Megahit.

**Supplementary Figure S1** Chromatograms of GC for analytical standards (A) and ruminal samples in LRDS group (B), MRDS group (C), and HRDS group (D).

**Supplementary Figure S2** Chromatograms of HPLC for analytical standards (A) and ruminal samples in LRDS group (B), MRDS group (C), and HRDS group (D).

**Supplementary Figure S3** The scaftigs length distribution for all samples.

**Supplementary Table S1** The summary statistics for Multiple_Megahit

| Sample | Contigs | Contigs bases(bp) | N50(bp) | N90(bp) | Max(bp) | Min(bp) |
| --- | --- | --- | --- | --- | --- | --- |
| L1 | 1483345 | 1082994230 | 771 | 365 | 203893 | 300 |
| L2 | 1839058 | 1297420351 | 764 | 371 | 222511 | 300 |
| L3 | 1704347 | 1250780852 | 784 | 370 | 248947 | 300 |
| L4 | 1403991 | 1043809681 | 798 | 369 | 385464 | 300 |
| L5 | 1638104 | 1145925846 | 736 | 365 | 185441 | 300 |
| L6 | 1779697 | 1244180507 | 745 | 369 | 170896 | 300 |
| M1 | 1442258 | 1111610750 | 869 | 376 | 179775 | 300 |
| M2 | 1590596 | 1225326385 | 875 | 379 | 359603 | 300 |
| M3 | 1482277 | 1097977822 | 804 | 369 | 165511 | 300 |
| M4 | 1506302 | 1127061798 | 809 | 372 | 196780 | 300 |
| M5 | 1498662 | 1172057181 | 879 | 376 | 316385 | 300 |
| M6 | 1767302 | 1244082982 | 743 | 367 | 474504 | 300 |
| H1 | 1580876 | 1170206901 | 793 | 369 | 289603 | 300 |
| H2 | 1627670 | 1175867469 | 780 | 369 | 245888 | 300 |
| H3 | 1834887 | 1301368290 | 743 | 365 | 221282 | 300 |
| H4 | 1289458 | 959415054 | 809 | 368 | 354197 | 300 |
| H5 | 1583842 | 1225973588 | 870 | 377 | 338126 | 300 |
| H6 | 1084290 | 820620569 | 846 | 370 | 368356 | 300 |
| Megahit_Mix | 11440886 | 7692818675 | 714 | 369 | 62141 | 300 |

**Supplementary Figure S1** Chromatograms of GC for analytical standards (A) and ruminal samples in LRDS group (B), MRDS group (C), and HRDS group (D).
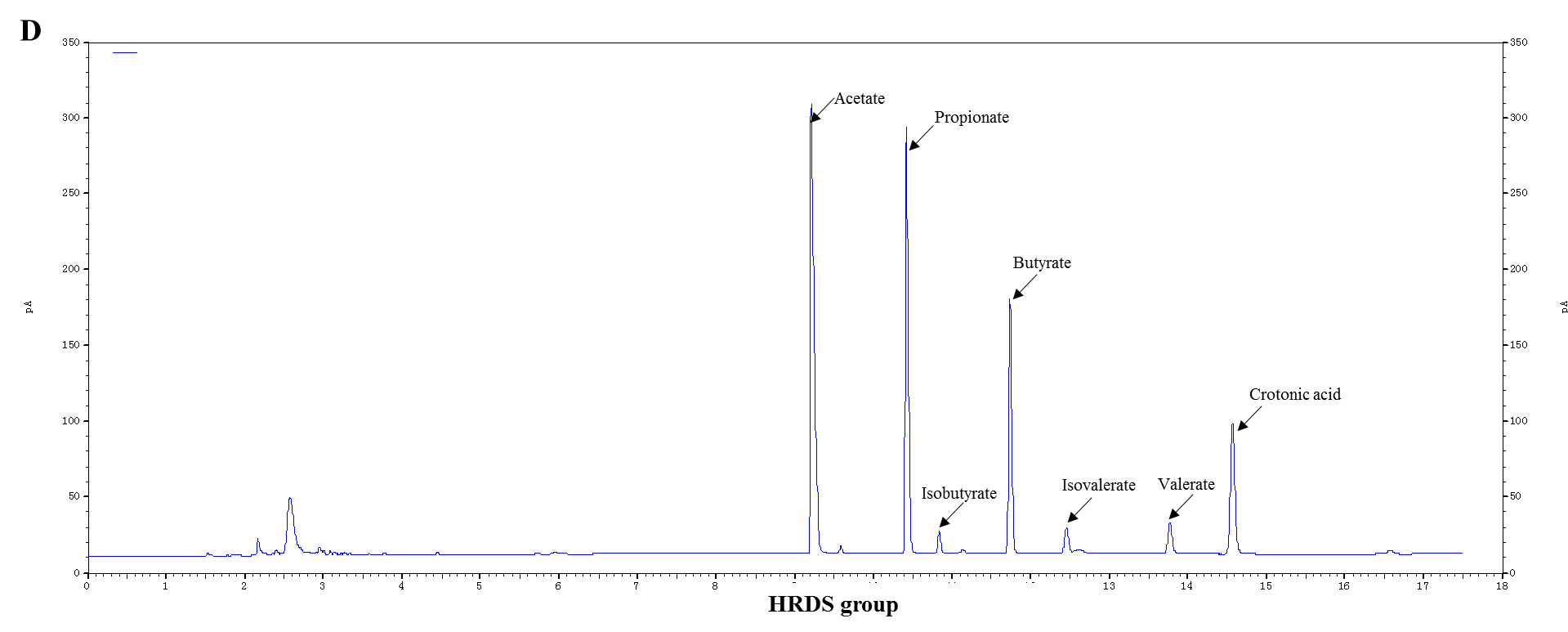

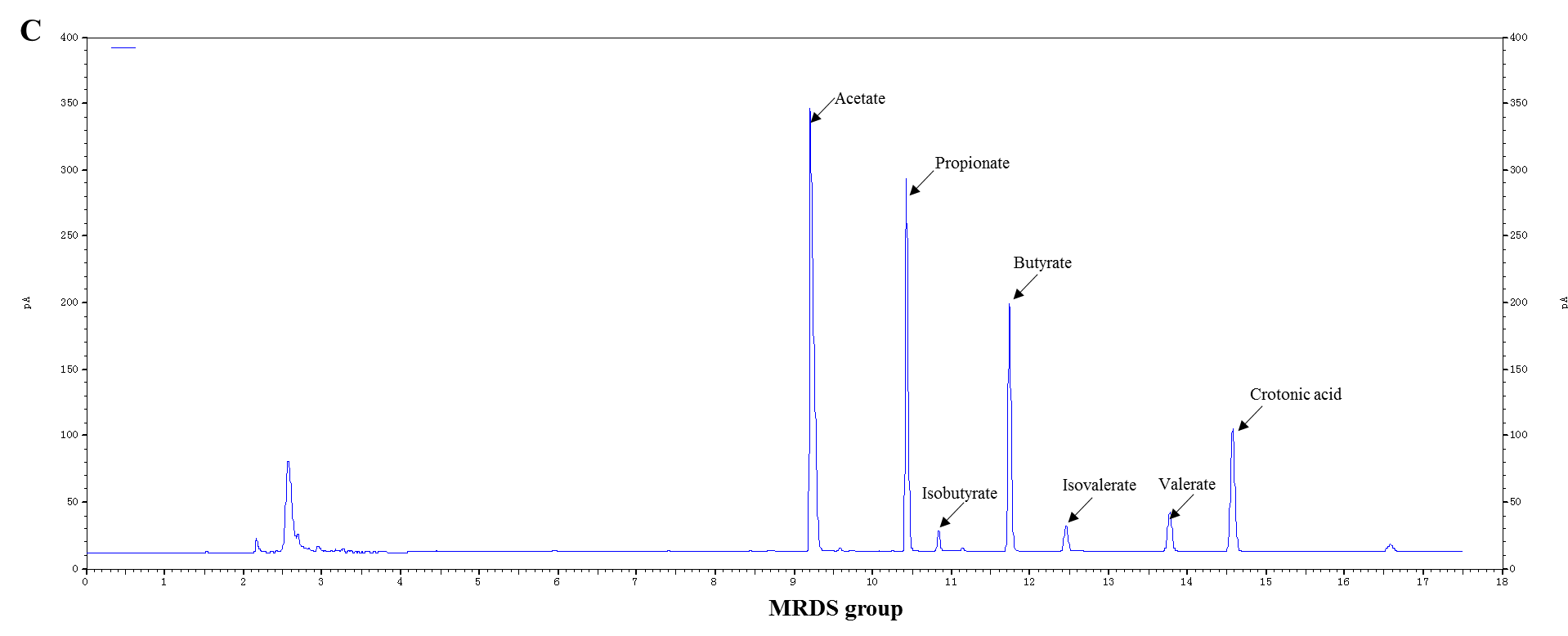

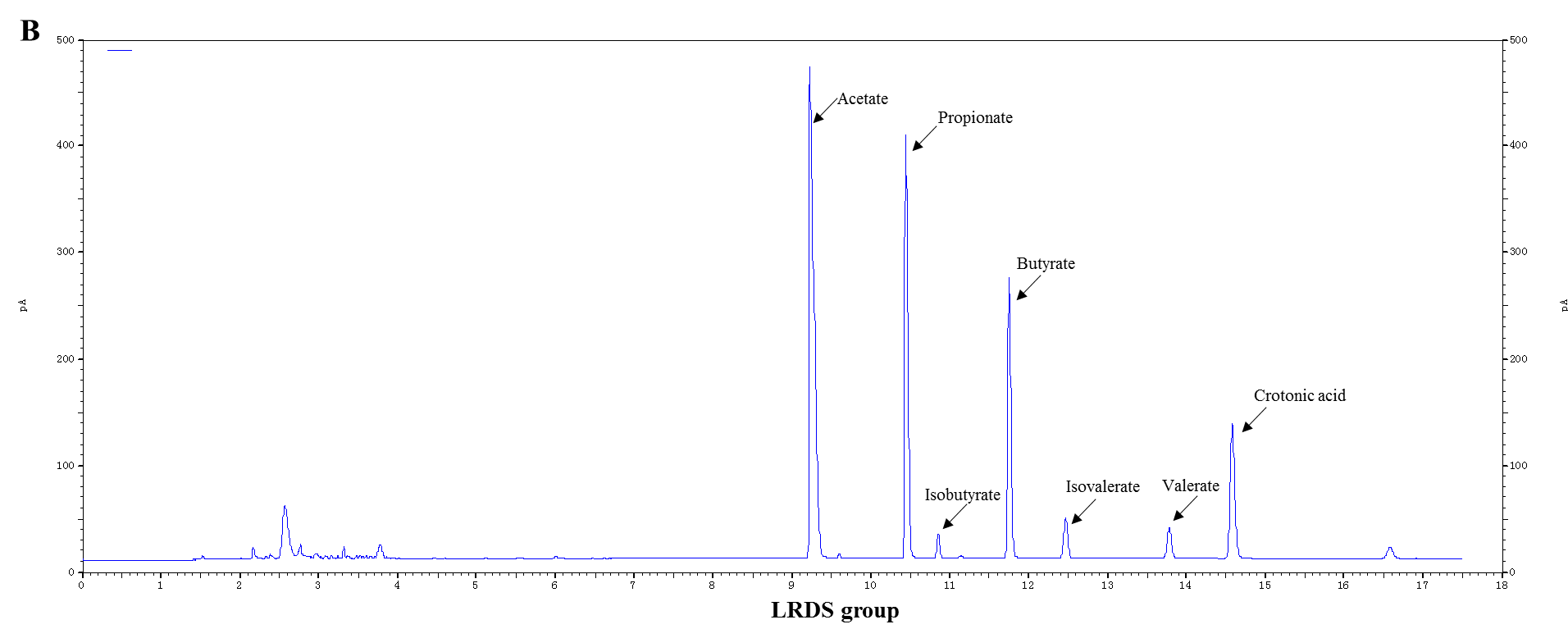

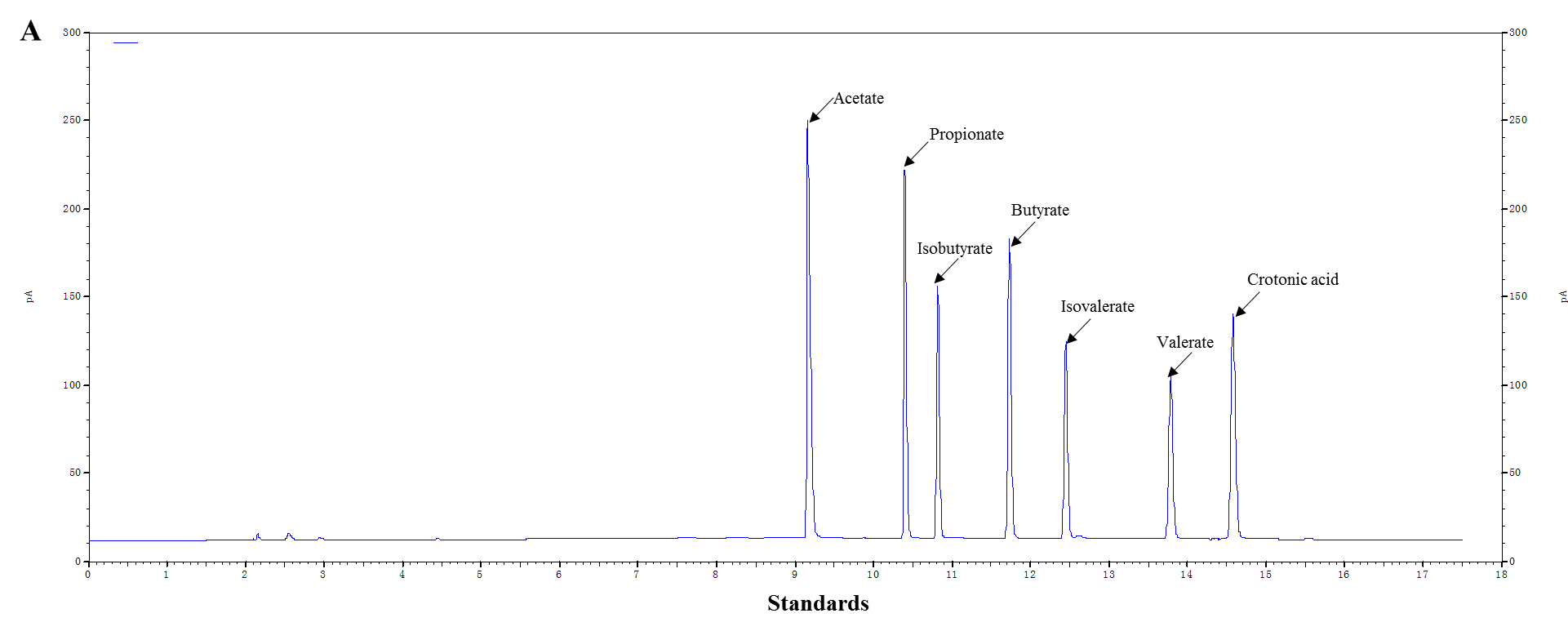


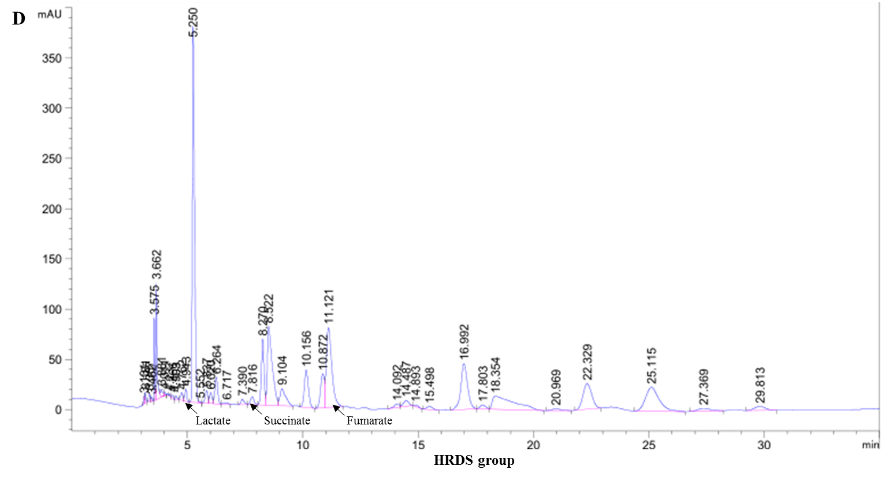

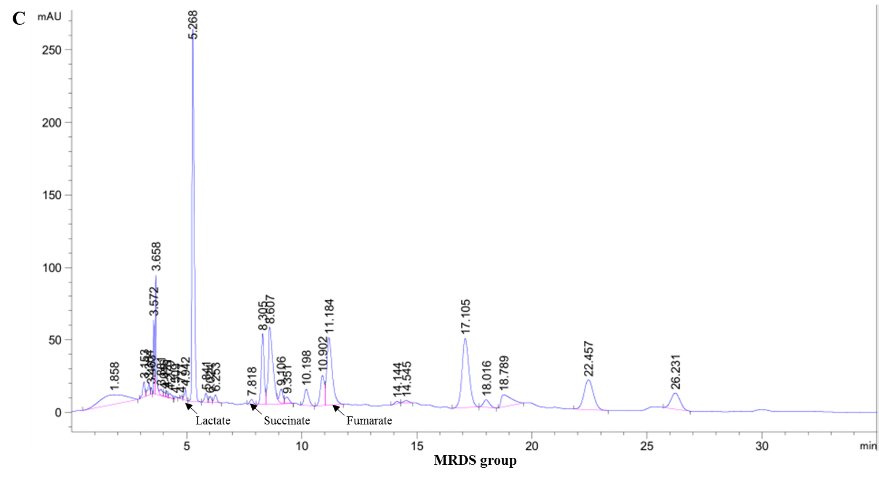

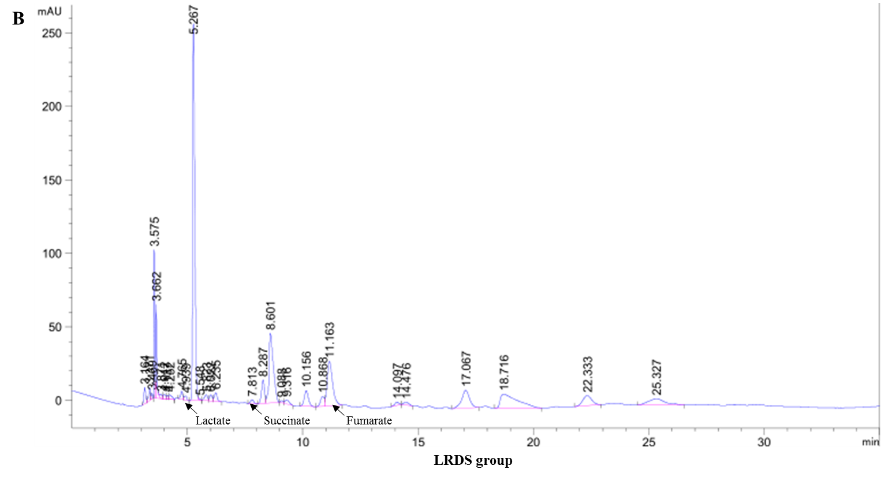

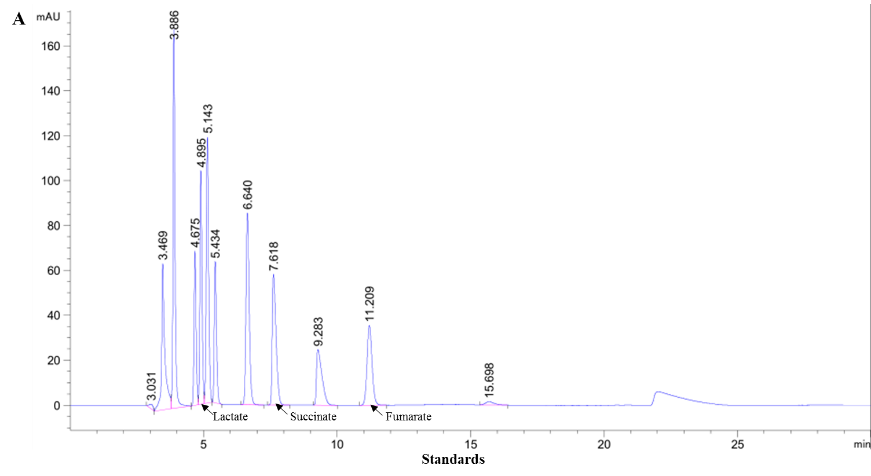
**Supplementary Figure S2** Chromatograms of HPLC for analytical standards (A) and ruminal samples in LRDS group (B), MRDS group (C), and HRDS group (D).


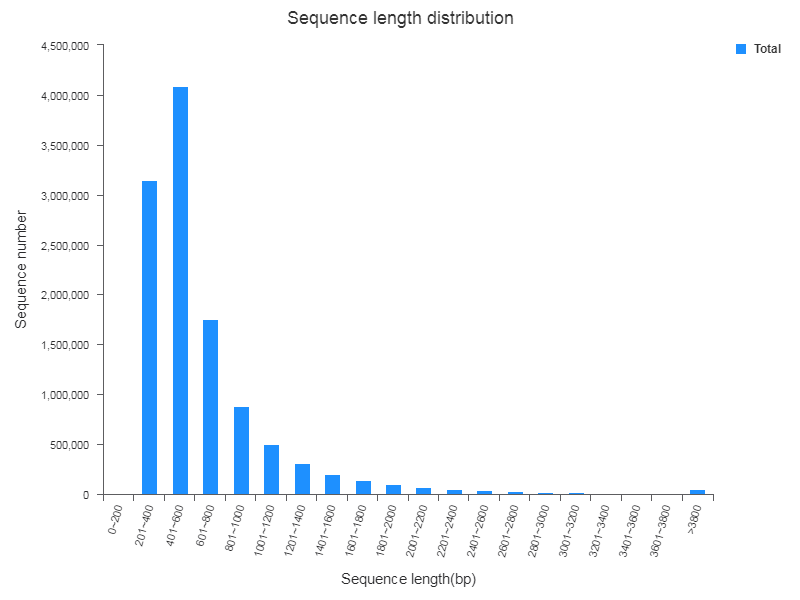
**Supplementary Figure S3** The scaftigs length distribution for all samples.
